# Temperament Traits as Key Modulators of Depression and Anxiety in Hyperthyroidism: Implications for Personalized Treatment

**DOI:** 10.7759/cureus.81503

**Published:** 2025-03-31

**Authors:** Deniz Alci, Mustafa Eroglu, Mehmet Asik

**Affiliations:** 1 Psychiatry, Balıkesir University, School of Medicine, Balıkesir, TUR; 2 Endocrinology, Diabetes, and Metabolism, Balıkesir University, School of Medicine, Balıkesir, TUR; 3 Endocrinology, Diabetes, and Metabolism, Bodrum American Hospital, Muğla, TUR

**Keywords:** anxiety, depression, endocrine psychiatry, hads scale, hyperthyroidism, mood disorders, personalized treatment, psychosomatic medicine, temperament traits, thyroid dysfunction

## Abstract

Introduction

The relationship between thyroid hormone dysfunction and psychiatric symptoms is complex, with hyperthyroidism frequently presenting alongside mood and anxiety disorders. Temperament traits, enduring psychological characteristics, may significantly influence the clinical manifestation of depression and anxiety in hyperthyroid patients, yet they remain understudied. This study aimed to determine whether specific temperament traits in hyperthyroid individuals correlate with anxiety and depressive symptoms and explore their implications for personalized clinical management.

Methods

A total of 59 patients diagnosed with hyperthyroidism and 40 healthy controls participated in this study. All participants underwent clinical assessment, thyroid function testing (thyroid-stimulating hormone (TSH), T3, T4), and completed the Hospital Anxiety and Depression Scale (HADS) and the Temperament Evaluation of Memphis, Pisa, Paris, and San Diego Autoquestionnaire (TEMPS-A). Demographic and clinical parameters, including age, gender, body mass index (BMI), blood pressure, smoking status, and family history, were also documented. Statistical analyses included Mann-Whitney U tests for group comparisons, Spearman's correlation analysis for relationships between temperament traits and psychiatric symptoms, and generalized linear models (GLM) to identify predictors of depressive symptoms.

Results

Hyperthyroid patients exhibited significantly higher anxiety scores compared to controls (p<0.001), whereas depressive symptoms, although elevated, did not reach statistical significance (p>0.05). Temperament assessment revealed significantly increased depressive, cyclothymic, irritable, and anxious traits in patients compared to controls (p<0.001). Hyperthymic temperament showed no significant difference between groups. A notable positive correlation emerged between irritable temperament and increased depressive symptoms (r=0.635, p<0.001). Depressive temperament significantly predicted higher depression scores, while hyperthymic temperament appeared protective, associated with reduced depressive symptoms (p<0.05).

Conclusions

These findings highlight the importance of temperament traits as critical modulators of psychiatric outcomes in hyperthyroidism. Irritable and depressive temperaments may increase vulnerability to depressive disorders, whereas hyperthymic temperament could offer resilience. Integrating temperament assessment into clinical practice may enhance diagnostic precision and inform personalized therapeutic approaches, improving patient outcomes and quality of life.

## Introduction

The thyroid gland's influence on mental processes has led to extensive research on the correlation between thyroid hormone abnormalities and psychiatric symptoms and disorders. Hormonal systems, particularly thyroid hormones, significantly influence social functioning through their regulatory effects on emotional regulation, social interactions, cognitive processes, and specific behavioral traits such as irritability and impulsivity. Dysregulation in thyroid hormones can impair emotional stability, increase irritability, alter cognitive clarity, and negatively influence interpersonal interactions, ultimately affecting social relationships and overall quality of life [1-3]. This enables the exploration of neurochemical models proposing that thyroid hormones may function as neurochemical biomarkers, particularly considering that imbalances in the hypothalamic-pituitary-thyroid axis contribute substantially to emotional instability [1]. Investigating biomarkers linked to particular psychopathological symptoms or biobehavioral traits, specifically temperaments, offers significant benefits for the early detection of disorders. Previous research has identified neurotrophic factors, such as brain-derived neurotrophic factor (BDNF) and nerve growth factor (NGF), as biomarkers associated with temperament traits like emotional instability and anxiety-proneness [4].

Thyroid hormone disorders represent a category of diseases necessitating the incorporation of psychosocial and cognitive elements into both diagnosis and treatment. Additionally, the overall functioning of patients is influenced by mental health issues stemming from endocrine origins. Prior research indicates that variations in thyroid hormone levels correlate with emotional instability, impulsivity, and cognitive dysfunction. This situation may heighten patients' vulnerability to psychosocial stressors [2].

Hyperthyroidism is a clinical condition characterized by elevated circulating levels of thyroid hormones [5]. Endogenous causes of hyperthyroidism include Graves' disease, toxic multinodular goiter, toxic adenoma, and silent thyroiditis. Graves' disease, the predominant etiology of hyperthyroidism, is an autoimmune condition characterized by the activation of thyroid-stimulating hormone (TSH) receptors by thyroid-stimulating antibodies, leading to the synthesis of thyroid hormones [5]. Elevated thyroid hormone levels augment catecholaminergic activity by increasing the quantity of beta-adrenergic receptors on the cell surface. The adrenergic symptoms resulting from hyperthyroidism, including palpitations, heat intolerance, sweating, tremors, eyelid retraction, and increased bowel movements, represent some of the most prevalent clinical manifestations of this condition [3]. Hypermetabolism leads to weight loss despite heightened appetite and may result in proximal muscle weakness, atrial fibrillation, and heart failure [6-8]. Furthermore, a diverse array of psychiatric symptoms may manifest, including anxiety to severe psychosis [9].

In cases of hyperthyroidism, psychiatric manifestations may occasionally serve as the initial indicators for the identification of hormone imbalances. Research investigating the correlation between hyperthyroidism and psychiatric symptoms such as depression and anxiety has yielded varying results [10]. This inconsistency is primarily related to variations in the manifestations and severity of psychiatric symptoms across different studies, as well as differences in treatment approaches and patient characteristics. Despite the absence of definitive evidence linking hyperthyroidism to a specific psychiatric disorder, thyroid hormones are recognized to induce various psychiatric symptoms. Thyroid hormones have been demonstrated to increase the level of alertness and mental activity, as well as accelerate cognitive processes such as thought associations, information processing speed, and mental responsiveness [11]. Anxiety, irritability, psychomotor agitation, insomnia, difficulty concentrating, and increased excitability of the sympathetic aspect of the autonomic nervous system can be induced by an increase in thyroid hormone concentration [12].

Depressive and anxiety symptoms may manifest as a result of diminished serotonergic and noradrenergic activity in the context of thyroid hormone deficiency (hypothyroidism) [13]. In contrast, in hyperthyroidism, neurotransmitter imbalances occur as a result of the disruption of homeostatic regulation. For instance, animal models with hyperthyroidism have been shown to exhibit reduced levels of dopamine and norepinephrine in the cerebral cortex [14]. Clinical applications indicate that T3 supplementation enhances the efficacy of antidepressants in treatment-resistant depression, and attaining a euthyroid state in hyperthyroid patients improves mood symptoms [15]. Consequently, an excess or deficit of thyroid hormones may facilitate the onset of depression, anxiety, or agitation by impairing the regulation of monoamine systems [13-15]. Although hyperthyroid patients initially experience excessive anxiety and excitement due to heightened adrenergic activity, chronic hyperthyroidism may lead to persistent emotional instability and vulnerability to mood disturbances. Previous studies suggest that hyperthyroid patients exhibit elevated temperamental traits such as depressive, anxious, and irritable temperaments, which are linked to long-term psychiatric vulnerability and poorer psychosocial outcomes [13,16].

According to research on the shared mechanism between psychiatric disorders and thyroid hormone disorders, fluctuations in the levels of neurotrophins, including NGF and BDNF, in thyroid dysfunction are similar to those observed in psychiatric disorders [4]. The convergence in biological processes suggests that certain temperamental traits observed in hyperthyroid individuals may be associated with a mental-physical phenomenon linked to an underlying endocrinological pathology.

The objective of this study is to determine whether the psychosocial symptoms observed in hyperthyroid cases are categorized within a specific temperament and to investigate the correlation between these temperamental characteristics and the severity of anxiety and depression.

## Materials and methods

Study design and participants

This cross-sectional study was conducted at the Endocrinology Outpatient Clinic of Çanakkale 18 Mart University Hospital between June 2016 and January 2017. Participants were recruited using consecutive sampling during routine outpatient visits. The study included 59 patients aged 18 to 65 years who were newly diagnosed with hyperthyroidism, with no history of psychiatric disorders or treatments, along with a control group of 40 healthy individuals recruited from the Internal Medicine Outpatient Clinic who were not diagnosed with any diseases upon examination.

A priori power analysis was conducted using G*Power software (version 3.1 Heinrich-Heine-Universität Düsseldorf, Düsseldorf, Germany), indicating that a minimum of 98 participants was required to detect a medium effect size (d=0.5) with 80% power and a 5% significance level. The total sample of 99 participants satisfied this requirement, indicating adequate statistical power for the primary analyses conducted in this study.

Prior to inclusion in the study, all participants underwent a clinical evaluation by an endocrinologist, and thyroid function tests (T3, T4, TSH, and anti-thyroid antibody levels) were measured. All participants, including both hyperthyroid patients and healthy controls, completed the Hospital Anxiety and Depression Scale (HADS) and the Temperament Evaluation of Memphis, Pisa, Paris, and San Diego Autoquestionnaire (TEMPS-A). Demographic and clinical data, including age, gender, body mass index (BMI), systolic and diastolic blood pressure, smoking status, and family history of coronary artery disease, were collected for all participants. All clinical and laboratory parameters, including thyroid hormone levels and blood pressure, were measured once during the initial assessment. No repeated measurements were taken.

Exclusion criteria

Participants with the specified conditions were excluded from the study: individuals with a formal psychiatric diagnosis or a history of psychiatric medication use were excluded. Participants with subclinical psychiatric symptoms (e.g., mild anxiety or depressive symptoms) who had not received a clinical diagnosis or treatment were excluded. Individuals with a history of malignancy, pregnant or breastfeeding women, and patients exhibiting physical or mental functional disabilities that hinder their ability to complete the scales are also excluded.

Acquisition of data procedure

The participants' data were documented following the assessment conducted by specialized physicians in the endocrinology clinic. Thyroid function tests were analyzed using routine biochemical techniques in the hospital laboratory.

The BMI was determined by dividing each participant's weight in kilograms by the square of their height in meters (kg/m²). Systolic and diastolic blood pressure readings were obtained using a mercury sphygmomanometer while the subject was seated, following a minimum of five minutes of rest.

The scales were completed by the participants themselves to assess their psychiatric conditions. The study protocol adhered to ethical guidelines, and ethical approval was obtained from the Çanakkale Onsekiz Mart University Clinical Research Ethics Committee (Approval No: 2015-11/05). Informed consent was acquired from all participants.

In addition to demographic data, we specifically examined the following clinical indicators in each participant: age, BMI, medication use, thyroid function tests (T3, T4, TSH, and anti-thyroid antibodies), and psychiatric/temperament measures (HADS and TEMPS-A).

Psychometric measures

Hospital Anxiety and Depression Scale (HADS)

The HADS was developed by Zigmond and Snaith (1983) and adapted into Turkish by Aydemir et al. [17,18]. It is a self-report scale designed to screen for anxiety and depression symptoms in individuals with physical illnesses. The scale consists of 14 items, with odd-numbered items assessing anxiety and even-numbered items assessing depression. Each item is scored on a 0-3 scale, with a cutoff score of 10 for the anxiety subscale and 7 for the depression subscale. The internal consistency coefficients of the scale were reported as 0.85 for the anxiety subscale and 0.78 for the depression subscale.

Temperament Evaluation of Memphis, Pisa, Paris, and San Diego Autoquestionnaire (TEMPS-A)

The TEMPS-A is a self-assessment questionnaire developed to evaluate temperamental traits. It examines temperament dimensions across five primary factors: depressive, cyclothymic, hyperthymic, irritable, and anxious [19]. The TEMPS-A consists of 99 items, with subscales distributed as follows: Depressive (21 items), Cyclothymic (19 items), Hyperthymic (20 items), Irritable (18 items), and Anxious (21 items). Items are rated true/false, and the total score for each temperament is calculated by summing the number of items endorsed as "true" within each dimension. Higher scores indicate a stronger expression of the respective temperamental trait. Participants complete the scale by selecting statements that best describe their general lifelong characteristics. The Turkish validity and reliability study was conducted by Vahip et al. [20]. In this study, Cronbach's alpha coefficients were reported as 0.77 for depressive, 0.78 for cyclothymic, 0.68 for hyperthymic, 0.71 for irritable, and 0.85 for anxious temperament.

The five temperament dimensions are defined as follows: A depressive temperament is characterized by pessimism, low energy, self-doubt, and a tendency to worry. Cyclothymic temperament involves mood swings, alternating periods of high and low energy, and affect. Hyperthymic temperament includes traits such as optimism, high energy, sociability, and overconfidence. Irritable temperament features a low tolerance for frustration, quick temper, and interpersonal conflict. Anxious temperament is defined by chronic worry, vigilance, and fearfulness.

Statistical analysis

All statistical analyses were conducted using IBM SPSS Statistics for Windows, Version 22 (Released 2013; IBM Corp., Armonk, New York). Descriptive statistics were used to summarize demographic and clinical characteristics. The Shapiro-Wilk test was used to assess normality. For group comparisons, an independent samples t-test was used for normally distributed variables, and the Mann-Whitney U test was used for non-normally distributed variables. The chi-square test was used for categorical data. Correlations between temperament traits and psychiatric symptoms were assessed using Spearman's rank correlation coefficient. To identify predictors of depression and anxiety scores, a generalized linear model (GLM) regression analysis was performed. A p-value < 0.05 was considered statistically significant.

## Results

The research aimed to investigate the impact of temperament traits on depression and anxiety in individuals with hyperthyroidism. The study had 99 participants, with 59.6% (n=59) belonging to the "Patient" group diagnosed with hyperthyroidism and 40.4% (n=40) from the "Control" group, which included healthy individuals without thyroid illness. The features of the patient group are presented in Tables 1, 2.

**Table 1:** Evaluation of Participants' Characteristics by Groups 1t: Student's t-test; 2χ²: Yates corrected chi-square test; 3χ²: Pearson chi-square test; *p < 0.05; **p < 0.01. Quantitative data are presented as a minimum–maximum and mean ± standard deviation, while qualitative data are shown as numbers (%). BMI: Body Mass Index; SBP: Systolic Blood Pressure; DBP: Diastolic Blood Pressure; CAD: Coronary Artery Disease.

Variable		Patient (n=59)	Control (n=40)	Total (N=99)	t / χ^2^	p-value
Age (years)	Min – Max	21-65	23-55	21-65	^1^2.295	0.032*
	Mean ± SD	40.54±12.03	35.70±8.94	38.59±11.10		
Age Group	≤35 years	20 (33.9)	26 (65)	46 (46.5)	^2^8.062	0.005**
	>35 years	39 (66.1)	14 (35)	53 (53.5)		
Gender	Female	41 (69.5)	31 (77.5)	72 (72.7)	^2^0.420	0.517
	Male	18 (30.5)	9 (22.5)	27 (27.3)		
BMI (kg/m^2^)	Min-Max	15.62-37.86	18.87-31.99	15.62-37.86	^1^0.015	0.988
	Mean ± SD	23.89±3.95	23.88±3.20	23.88±3.65		
BMI Category	Underweight	3 (5.1)	0 (0)	3 (3)	^3^2.282	0.516
	Normal	41 (69.5)	30 (75)	71 (71.7)		
	Overweight	11 18.6)	8 (20)	19 (19.2)		
	Obese	4 (6.8)	2 (5)	6 (6.1)		
SBP (mmHg)	Min-Max	90-164	86-160	86-164	^1^2.874	0.005**
	Mean ± SD	121.20±15.99	112.35±13.50	117.63±15.59		
DBp (mmHg)	Min-Max	50-100	50-90	50-100	^1^2.706	0.008**
	Mean ± SD	74.02±8.94	69.30±7.82	72.11±8.78		
Smoking	Yes	19 (32.2)	10 (25)	29 (29.3)	^2^0.597	0.440
	No	40 (67.8)	30 (75)	70 (70.7)		
Family History of CAD	Yes	9 (15.3)	4 (10)	13 (13.1)	^2^0.208	0.648
	No	50 (84.7)	36 (90)	86 (86.9)		

**Table 2:** Distribution of Characteristics in the Patient Group (n=59) Quantitative data are presented as minimum–maximum and mean ± standard deviation (median) due to high variance, while qualitative data are shown as numbers (%). HT: Hypertension; TSH: Thyroid-Stimulating Hormone; T4 (ng/dL): Thyroxine, measured in nanograms per deciliter; T3 (ng/dL): Triiodothyronine, measured in nanograms per deciliter.

Characteristics of the Patient Group (n=59)		n (%)
History of HT	Yes	3 (5.1)
	No	56 (94.9)
Other Diseases	Yes	6 (10.2)
	No	52 (89.8)
Medication Use	Yes	18 (30.5)
	No	41 (69.5)
TSH (mIU/L)	Min-Max	0.001-0.280
	Mean ± SD (Median)	0.020±0.051 (0.005)
T4 (ng/dL)	Min-Max	0.91-45.57
	Mean ± SD (Median)	4.24±5.85 (3.29)
T3 (ng/dL)	Min-Max	2.70-26.98
	Mean ± SD (Median)	11.61±5.61 (11.64)

Cronbach's α internal consistency coefficients for the subdimensions of the TEMS-A and HADS scales indicate that the scale possesses excellent internal dependability overall. The Cronbach's α internal consistency coefficient for the TEMS-A scale was determined to be notably high at 0.937. Table 3 presents the score ranges for the five distinct temperament subdimensions assessed by the TEMS-A scale.

**Table 3:** Distribution of TEMPS-A Temperament and Hospital Anxiety and Depression (HADS) Scales Quantitative data are presented as minimum–maximum and mean ± standard deviation (median) due to high variance. Cronbach's α: Internal Consistency Coefficient; TEMPS-A: Temperament Evaluation of Memphis, Pisa, Paris, and San Diego Autoquestionnaire; HADS: Hospital Anxiety and Depression Scale.

Scales	Subdimensions	Number of Items	Min-Max	Mean±SD (Median)	Cronbach α
TEMS-A	Depressive temperament	19	0-17	6.79±4.19 (6)	0.828
	Cyclothymic temperament	19	0-18	8.29±5.13 (8)	0.878
	Hyperthymic temperament	20	0-20	10.07±4.79 (10)	0.838
	Irritable temperament	18	0-16	4.65±4.20 (4)	0.869
	Anxious temperament	24	0-24	8.01±6.06 (7)	0.897
HADS	Anxiety	8	1-19	8.14±4.58 (7)	0.843
	Depression	8	0-18	6.18±4.27 (5)	0.795

When the responses to the 100th item of the TEMPS-A, which asks participants to select the temperament description that best reflects their lifelong characteristics, were examined, 29.3% (n=29) reported experiencing emotional fluctuations and numerous ups and downs (consistent with cyclothymic features); 21.2% (n=21) selected the statement "I am a calm person," which is one of the response options in item 100 but is not part of the five standard TEMPS-A temperament dimensions. Additionally, 18.2% (n=18) identified themselves as totally energetic and cheerful (hyperthymic-like), 12.1% (n=12) as easily irritable, 10.1% (n=10) as persistently anxious, and 9.1% (n=9) as experiencing long-term depressive mood throughout their lives.

In terms of depressive temperament, the patient group had a mean score of 8.29±4.21 (median: 8), whereas the control group had a mean score of 4.58±3.07 (median: 4). The scores for depressive temperament, cyclothymic temperament, irritable temperament, and anxious temperament in the patient group were statistically significantly higher compared to the control group (p<0.001). No statistically significant difference was seen between the groups for hyperthymic temperament scores (p>0.05). The mean score for the HAD-Anxiety subdimension in the patient group was 6.49±3.59 (median: 7), while the control group had a mean score of 1.95±0.22 (median: 2). The anxiety level was statistically considerably elevated in the patient group (p<0.001). In the HADS-Depression subscale, the patient group had higher depression scores; however, this disparity did not attain statistical significance (p>0.05). Table 4 presents a comparison of anxiety levels, depression levels, and temperament traits between the groups.

**Table 4:** Evaluation of TEMS-A Temperament and HADS Scores by Groups **p<0.01; Quantitative data are presented as mean ± standard deviation along with the median since they do not follow a normal distribution. Z: Mann-Whitney U test; TEMPS-A: Temperament Evaluation of Memphis, Pisa, Paris, and San Diego Autoquestionnaire; HADS: Hospital Anxiety and Depression Scale.

Scales	Subdimensions	Patient (n=59)	Control (n=40)	Z	p-value
		Mean ± SD (Median)	Mean ± SD (Median)		
TEMS-A	Depressive temperament	8.29±4.21 (8)	4.58±3.07 (4)	-4.351	<0.001**
	Cyclothymic temperament	10.22±4.82 (11)	5.45±4.21 (5)	-4.511	<0.001**
	Hyperthymic temperament	9.92±4.91 (10)	10.3±4.65 (10)	-0.089	0.929
	Irritable temperament	5.93±4.55 (5)	2.75±2.70 (2)	-3.421	0.001**
	Anxious temperament	9.75±5.91 (10)	5.45±5.38 (3)	-3.623	<0.001**
HADS	Anxiety	6.49±3.59 (7)	1.95±0.22 (2)	-7.266	<0.001**
	Depression	6.93±4.74 (7)	5.08±3.21 (5)	-1.815	0.070

In the patient group, there were significant positive correlations between depressive, cyclothymic, irritable, and anxious temperament dimensions and depression (p<0.001). A significant correlation was found between irritable temperament and depression (r=0.635, p<0.001). Nonetheless, no significant correlation was identified between temperament dimensions and anxiety in the patient group (p>0.05). In the control group, there was a significant correlation between depressive and anxious temperaments and depression (p<0.05), whereas hyperthymic temperament exhibited an inverse correlation with depression (r=-0.385, p=0.014). Other temperament dimensions did not exhibit a significant correlation with anxiety or depression (p>0.05). Depressive, cyclothymic, irritable, and anxious temperament characteristics exhibited a direct correlation with depression levels in hyperthyroid patients; however, they did not show a significant correlation with anxiety. In the control group, depressive and anxious temperaments were correlated with depression, whereas hyperthymic temperament exhibited an inverse correlation with depression. In the control group specifically, none of the temperament dimensions showed a statistically significant correlation with anxiety levels (p>0.05), indicating that temperament traits were not associated with anxiety symptoms among healthy individuals. The findings indicate that specific temperament characteristics may be more closely linked to the development of depression in hyperthyroid patients (Table 5).

**Table 5:** Correlation Evaluation of TEMS-A Temperament and HAD Scale Scores by Groups *p<0.05; **p<0.01; r: Spearman's Rho Correlation Coefficient; TEMPS-A: Temperament Evaluation of Memphis, Pisa, Paris, and San Diego Autoquestionnaire; HADS: Hospital Anxiety and Depression Scale

Groups	TEMS-A	HADS			
		Anxiety		Depression	
		r	p-value	r	p-value
Patient (n=59)	Depressive temperament	0.073	0.584	0.592	<0.001**
	Cyclothymic temperament	-0.077	0.561	0.505	<0.001**
	Hyperthymic temperament	0.122	0.357	-0.082	0.535
	Irritable temperament	0.141	0.286	0.635	<0.001**
	Anxious temperament	0.072	0.588	0.453	<0.001**
Control (n=40)	Depressive temperament	-0.126	0.440	0.380	0.016*
	Cyclothymic temperament	-0.110	0.500	0.252	0.117
	Hyperthymic temperament	-0.160	0.325	-0.385	0.014*
	Irritable temperament	-0.131	0.421	0.279	0.081
	Anxious temperament	-0.115	0.480	0.388	0.013*

A notable difference was observed between hyperthymic temperament and gender within the patient group (p=0.038; p<0.05). Male patients exhibited higher scores in hyperthymic temperament compared to female patients (male: 11.94±4.89; female: 9.02±4.70). A significant positive correlation exists between TSH levels and anxious temperament (r=0.276, p=0.034; p<0.05). This finding indicates that higher TSH levels are associated with an increased tendency toward anxious temperament. The findings suggest that gender may influence hyperthymic temperament in hyperthyroid patients, that elevated diastolic blood pressure correlates with irritable temperament, and that TSH levels could be a significant factor in anxious temperament. However, no significant correlation was found between BMI and irritable temperament (r=0.197, p=0.134) (Table 6).

**Table 6:** Evaluation of TEMS-A Scale Scores According to Patient Group Characteristics Due to the non-normal distribution of quantitative data, both mean ± standard deviation and median values were reported. Statistical comparisons were conducted using the Mann-Whitney U test (Z-values) and Spearman's rank correlation coefficient (r-values) where appropriate. TEMPS-A: Temperament Evaluation of Memphis, Pisa, Paris, and San Diego Autoquestionnaire; BMI: Body Mass Index; SBP: Systolic Blood Pressure; DBP: Diastolic Blood Pressure; CAD: Coronary Artery Disease; TSH: Thyroid-Stimulating Hormone; T3/T4: Triiodothyronine/Thyroxine; r: Spearman Rho Correlation Coefficient; Z: Mann-Whitney U test Statistic; p: Significance Level; p < 0.05: Statistically Significant.

Patient Group Characteristics (n=59)		Depressive Temperament	Cyclothymic Temperament	Hyperthymic Temperament	Irritable Temperament	Anxious Temperament
Age (years)	r	-0.091	-0.144	0.046	-0.066	-0.032
	p-value	0.493	0.276	0.727	0.618	0.811
Age group	≤35 years	8.25±4.04 (9)	11.55±4.94 (13)	9.75±4.51 (10)	6.50±4.76 (5.5)	10.50±6.35 (9.5)
	>35 years	8.31±4.34 (8)	9.54±4.68 (10)	10.00±5.16 (10)	5.64±4.47 (5)	9.36±5.72 (10)
	Z	-0.048	-1.470	-0.241	-0.627	-0.698
	p	0.962	0.142	0.810	0.530	0.485
Gender	Female	8.49±4.59 (9)	9.83±4.98 (10)	9.02±4.70 (9)	5.59±4.70 (4)	10.07±5.89 (9)
	Male	7.83±3.24 (8)	11.11±4.43 (11)	11.94±4.89 (12)	6.72±4.21 (6)	9.00±6.07 (11)
	Z	-0.636	-0.983	-2.080	-1.116	-0.371
	p	0.525	0.326	0.038*	0.264	0.711
BMI (kg/m^2^)	r	0.096	0.059	0.094	-0.199	-0.029
	p	0.471	0.659	0.480	0.130	0.830
SBP (mmHg)	r	0.196	0.069	0.072	0.058	0.032
	p	0.137	0.601	0.590	0.660	0.807
DBP (mmHg)	r	0.196	0.197	0.137	0.292	0.128
	p	0.138	0.135	0.299	0.025*	0.335
Smoking status	Yes	8.16±3.92 (8)	10.79±4.33 (11)	10.47±5.19 (11)	6.42±4.49 (7)	9.79±5.39 (10)
	No	8.35±4.38 (8.5)	9.95±5.07 (10.5)	9.65±4.81 (10)	5.70±4.61 (5)	9.73±6.21 (9)
	Z	-0.098	-0.521	-0.618	-0.660	-0.138
	p	0.922	0.603	0.536	0.509	0.890
Family history of CAD	Yes	8.67±3.84 (8)	9.00±4.18 (8)	10.56±3.68 (10)	5.56±4.13 (5)	10.67±3.61 (11)
	No	8.22±4.30 (8.5)	10.44±4.93 (11)	9.8±5.12 (10.5)	6.00±4.66 (5.5)	9.58±6.25 (9)
	Z	-0.264	-1.036	-0.254	-0.212	-0.644
	p	0.792	0.300	0.800	0.832	0.519
Other diseases	Yes	8.17±4.79 (6.5)	11.33±3.88 (12)	11.67±5.75 (13)	5.50±6.16 (3.5)	10.33±5.13 (12)
	No	8.30±4.19 (9)	10.09±4.93 (10)	9.72±4.83 (10)	5.98±4.40 (6)	9.68±6.04 (9)
	Z	-0.151	-0.579	-0.905	-0.390	-0.377
	p	0.880	0.563	0.365	0.696	0.706
Medication use	Yes	9.00±4.07 (9.5)	9.17±4.78 (10.5)	9.67±4.99 (10)	6.11±4.63 (5.5)	9.72±6.12 (10.5)
	No	7.98±4.27 (8)	10.68±4.82 (11)	10.02±4.93 (10)	5.85±4.57 (5)	9.76±5.90 (9)
	Z	-0.949	-1.007	-0.305	-0.248	-0.074
	p	0.343	0.314	0.760	0.804	0.941
TSH (mIU/L)	r	0.190	0.107	-0.197	0.149	0.276
	p	0.149	0.422	0.135	0.260	0.034*
T4 (ng/dL)	r	0.215	0.100	0.094	0.186	0.034
	p	0.103	0.453	0.480	0.159	0.797
T3 (ng/dL)	r	0.043	0.051	0.098	0.007	-0.108
	p	0.745	0.703	0.461	0.956	0.415

The GLM was employed to assess the impact of temperament characteristics on anxiety and depression in patients with hyperthyroidism. The suitability of the model, along with significance tests and coefficients, is presented below. Given the absence of any observed effect of temperament subdimensions on the anxiety subdimension, modeling was conducted solely on depression levels (Table 7).

**Table 7:** Predictors of HAD-Depression Score (Generalized Linear Model) *p<0.05; **p<0.01 SE: Standard Error; CI: Confidence Interval; BMI: Body Mass Index.

Patient Group Variables (n=59)	Estimate	SE	t	p-value	95% CI	
					Lower Limit	Upper Limit
Intercept	-7.567	2.698	-2.805	0.007*	-12.854	-2.279
Age group (<35 years vs. ≥35 years)	1.477	0.845	1.748	0.087	-0.179	3.133
BMI (kg/m^2^)	0.471	0.110	4.271	-	0.255	0.687
Medication use (Yes vs. No)	-1.885	0.878	-2.146	0.037*	-3.606	-0.163
Depressive temperament	0.371	0.126	2.935	0.005**	0.123	0.619
Hyperthymic temperament	-0.232	0.091	-2.542	0.014*	-0.411	-0.053
Irritable temperament	0.781	0.121	6.455	-	0.544	1.018
Anxious temperament	-0.214	0.100	-2.135	0.038*	-0.411	-0.018

This study developed a GLM utilizing a Gaussian (normal) distribution with an identity link function. The Gaussian-Identity model is utilized when the dependent variable is continuous, resembling classical linear regression.

The backward elimination method was employed for model selection. This method begins with the inclusion of all variables in the model, subsequently eliminating the least significant variables, characterized by high p-values, in a stepwise manner. At each step, the optimal model is chosen by evaluating the AIC (Akaike Information Criterion) and BIC (Bayesian Information Criterion) values of the model. Lower values of AIC and BIC signify improved model performance. The process persists until a model is established in which all remaining variables exhibit statistical significance (p<0.05) and the information criteria are optimized.

The final model, H₁, which incorporated all variables, was evaluated against the H₀ model, which contained only the constant term. The evaluation of the models revealed a deviance value of 1305.729 for the H₀ model, with an AIC of 354.157 and a BIC of 358.312. In contrast, the H₁ model exhibited a deviance of 448.539, an AIC of 305.114, and a BIC of 323.812. The notable reduction in the AIC and BIC values of the H₁ model (AIC: 354-305, BIC: 358-324) indicates an improved fit to the data, with the additional variables providing a meaningful contribution to the model. To further optimize the model, the backward elimination method was employed, systematically removing variables with the highest p-values. In the final stage, the optimal model was identified based on the AIC and BIC criteria, retaining the variables of age group, BMI, medication use, depressive temperament, hyperthymic temperament, irritable temperament, and anxious temperament. While the impact of the age group on the depression score was not statistically significant, it was included in the model due to its significance value being near 0.05, indicating a notable contribution to the model (p>0.05). The analysis of depression scores revealed that patients under 35 exhibited an average score 1.477 points higher than those aged 35 and older.

The average depression score for individuals using medication is 1.885 points lower compared to those not using medication. The reduction observed was statistically significant (p=0.037). A one-unit increase in BMI corresponds to an average increase of 0.471 points in the depression score. The observed increase was statistically significant (p<0.001).

Depressive temperament is a significant determinant of depression. An increase of one unit in the depressive temperament score corresponds to an average increase of 0.371 points in the depression score, with this effect being statistically significant (p=0.005). With each unit increase in the hyperthymic temperament score, the depression score decreases by an average of 0.232 points, a finding that is statistically significant (p=0.014).

An increase in irritable temperament correlates with a rise in depression scores. With each unit increase in the irritable temperament score, there is an average increase of 0.781 points in the depression score, a finding that is statistically significant (p<0.001). An increase in anxious temperament correlates with a decrease in depression scores. With each unit increase in the anxious temperament score, there is an average decrease of 0.214 points in the depression score, a finding that is statistically significant (p=0.038).

## Discussion

Despite research on psychiatric symptoms associated with thyroid dysfunction being prevalent in the literature, there is a deficiency in the investigation of enduring indicators, such as temperament, in hyperthyroid individuals. Temperament is a psychological characteristic that persists throughout an individual's life. This variable must be taken into account in the correlation between endocrine parameters and psychiatric symptoms. Literature indicates a strong correlation between hyperthyroidism and anxiety symptoms, with certain studies demonstrating that the prevalence of depression among patients with Graves' illness is markedly elevated compared to the control group [21]. Yuan et al. indicated that the Hamilton Anxiety and Hamilton Depression scores of patients with active hyperthyroidism are markedly elevated compared to those of healthy individuals [22]. According to a meta-analysis published in 2022 involving 15 studies and a total of 239,608 participants, individuals with hyperthyroidism were 1.7 times more likely to be diagnosed with depression than euthyroid individuals [23]. In this study, clinical indicators such as age, BMI, medication use, thyroid hormone levels (T3, T4, TSH), and temperament traits were examined in relation to depression and anxiety levels. Our investigation revealed that anxiety levels were significantly elevated in the hyperthyroidism group compared to the control group (HADS-Anxiety: 6.49±3.59 vs. 1.95±0.22; p<0.001), whereas the difference in HADS-Depression scores between the two groups did not reach statistical significance (6.93±4.74 vs. 5.08±3.21; p = 0.070).

The examination of the influence of several clinical indicators on depression levels in hyperthyroid individuals yields significant data. The investigation revealed no significant correlation between anxiety and subdimensions of temperament; hence, the assessment concentrated on levels of depression. This study found that depression scores in individuals under 35 were considerably elevated compared to those in patients aged 35 and older. This may be explained by the fact that younger patients may be more vulnerable to social and occupational stressors or that age-related physiological factors such as greater hormonal sensitivity could influence mood regulation differently across age groups. Meta-analysis results indicate that the association between hyperthyroidism and depression is applicable across all age demographics, in contrast to this study [23]. This discrepancy may be attributable to differences in study design, measurement tools, or sample size and warrants further investigation in future research with larger and more diverse cohorts. Nonetheless, the signs of depression in hyperthyroidism may vary across different age groups. The condition termed "apathic thyrotoxicosis" is commonly observed in elderly patients, where typical sympathetic nervous system manifestations (tremor, tachycardia, hyperactivity) may be absent, and unexplained weight loss, weakness, depression, or apathy may predominate [24]. Nonetheless, depression is not entirely uncommon among young patients; for instance, a follow-up study involving young women with Graves' disease indicated that while anxiety and depression scores, initially high, significantly improved post-treatment, they remained marginally elevated compared to controls even after 15 months [25].

The present study found that depression scores in participants on regular medication significantly decreased compared to those not using medication. Similarly, certain studies indicate that symptoms of depression and anxiety in hyperthyroid individuals may resolve within a few months of initiating treatment [26]. This swift enhancement suggests that mood also improves as the physiological impact of increased thyroid hormone levels subsides. Reports indicate that depression and anxiety scores markedly diminish as thyroid hormone levels normalize with adequate treatment, particularly in patients with early-stage Graves' illness [26]. This conclusion, aligned with the literature in our study, suggests that pharmaceutical use may have a protective impact in alleviating depressed symptoms.

The findings indicate that each unit increment in BMI correlates with an elevation in depression scores. This indicates that a higher BMI may correlate with depressive states. Moreover, depressive temperament correlates with elevated depression ratings with each incremental rise, highlighting the influence of temperament characteristics on depression. These findings underscore the significance of integrating parameters such as age, medication usage, BMI, and depressive mood in assessing the psychological condition of hyperthyroid patients.

Temperament characteristics in hyperthyroid patients are the subject of a restricted number of studies. Draganić-Gajić et al. (2008) showed that hyperthyroid people exhibit a heightened susceptibility to depressed, hypochondriacal, and histrionic personality traits, alongside elevated harm avoidance scores on the Cloninger scale [16]. This study also noted that hyperthyroid patients exhibited substantially higher levels of depressive, cyclothymic, irritable, and anxious temperament traits. Fukao et al. (2004) demonstrated that depressed personality traits endure even during hyperthyroidism remission and elevate the probability of relapse [13]. This study revealed that hyperthyroid people exhibited depressive, cyclothymic, irritable, and anxious temperament features at a markedly higher frequency than the control group. Upon examining the temperament states that most accurately characterize the subjects, 29.3% reported experiencing emotional lability, suggesting the neurochemical influence of hyperthyroid hormones, which have been shown to modulate neurotransmitter systems such as serotonin and norepinephrine that are implicated in mood regulation [27]. This corroborates the links between anxiety and depression documented in the literature. The absence of a significant difference in hyperthymic temperament between the two groups suggests that the energetic and enthusiastic traits of hyperthyroid patients do not sufficiently distinguish them from control individuals. This research indicates that the impact of hyperthyroid hormones is predominantly focused on emotional instability and hypersensitivity, encompassing depressive, cyclothymic, irritable, and anxious traits. This study suggests that clinical management of mood symptoms in hyperthyroid patients necessitates addressing both psychiatric disorders and temperament traits.

While hyperthyroidism was not directly linked to the degree of depression in this research, our results indicate that each unit increase in depressed temperament resulted in an average rise of 0.371 points in depression scores. Hyperthymic temperament was correlated with a notable reduction in depression scores, whereas hyperthyroid patients exhibiting prominent irritable temperament demonstrated elevated depression. This result may indicate a correlation between specific temperament traits and mood episodes, highlighting that existing temperament traits in patients with hyperthyroidism could signal a potential depressive episode, while hyperthymic traits may serve as a protective factor against depression. Multiple potential causes exist for the simultaneous occurrence of irritable temperament and depression in hyperthyroid patients: The direct actions of thyroid hormones on the neurological system may result in the manifestation of both symptom kinds. The decline in quality of life resulting from hyperthyroidism, chronic physical illness, and sleep difficulties may also promote the onset of a sad mood. A hyperthyroid patient exhibiting an irritable disposition may encounter difficulties in interpersonal connections while managing the physical manifestations of the condition, perhaps heightening the risk of depression by exacerbating feelings of isolation or powerlessness. Irritability and depression in hyperthyroidism are frequently interconnected, potentially perpetuating a reciprocal cycle [13]. Moreover, although hyperthymic temperament is more prevalent in men, the rise in anxious temperament corresponding with elevated TSH levels corroborates the influence of physiological alterations on temperament traits. These findings are significant as they indicate both biological and temperament-based factors may influence the onset of depression in hyperthyroid individuals. The robust correlation between irritable temperament and depression suggests that mood control mechanisms may operate differently in this patient population. This relationship, newly introduced in the literature, indicates that temperament traits must be included in the mental assessment of hyperthyroid patients, since these aspects may aid in predicting the likelihood of depression. Our research significantly enhances the comprehension of psychological symptoms linked to hyperthyroidism and informs the formulation of personalized treatment strategies.

Future studies should further explore both clinical and research dimensions of temperament traits and psychiatric outcomes in hyperthyroid patients. Clinically, incorporating temperament assessments routinely into endocrinology practice may facilitate personalized care, enabling early detection of patients at higher psychiatric risk, thus allowing timely multidisciplinary interventions. Collaboration among endocrinologists, psychiatrists, clinical psychologists, and primary care providers is essential to ensure comprehensive management. Such multidisciplinary teams could design individualized treatment plans that address hormonal imbalances, psychiatric conditions, and psychosocial stressors simultaneously, potentially improving patient outcomes and quality of life.

From a research perspective, longitudinal studies are critical for understanding the temporal relationships and causality between thyroid dysfunction, temperament traits, and psychiatric conditions. Future research should include larger sample sizes, multicenter designs, and diverse patient populations to enhance generalizability and to better understand the biopsychosocial mechanisms underlying mood and anxiety symptoms. Additionally, investigating specific biological markers (such as neurotransmitter and inflammatory markers) alongside psychosocial variables may provide deeper insights into the pathophysiology and guide more targeted therapeutic strategies.

Study limitations

This study offers valuable insights into the association between temperament traits and psychiatric symptoms in hyperthyroid patients; however, certain limitations should be acknowledged. First, the cross-sectional nature of the study precludes the establishment of causal relationships between temperament traits and psychiatric outcomes. Future longitudinal research is necessary to further investigate the directionality and long-term implications of these associations.

Second, the study was conducted in a single-center setting with a relatively limited sample size (59 hyperthyroid patients and 40 controls). Although the sample was carefully selected to minimize confounding factors, the findings may not be fully generalizable to broader populations. Multicenter studies with larger and more diverse cohorts would enhance the external validity of these results.

Third, the study relied on self-reported psychometric assessments, which may be subject to response bias. While validated scales were utilized to ensure methodological rigor, incorporating clinician-administered assessments in future studies could provide more objective evaluations of psychiatric symptoms.

Additionally, the relatively short data collection period constrained the ability to assess long-term treatment effects and adaptation mechanisms. Longitudinal follow-ups examining the persistence of psychiatric symptoms post-treatment would contribute to a more comprehensive understanding of the interplay between thyroid dysfunction and mental health.

Finally, despite controlling for factors such as age, BMI, and medication use, other biopsychosocial variables, including chronic stress, social environment, and quality of life, were not fully accounted for. Future studies should consider integrating a more extensive range of psychosocial parameters to provide a holistic perspective on the mental health implications of hyperthyroidism.

These limitations highlight the need for further research incorporating larger, multicenter, and longitudinal designs to validate and extend the present findings.

## Conclusions

This study elucidates the intricate relationship between temperament traits and depression in hyperthyroid patients, suggesting a need for re-evaluation of treatment approaches. The findings indicate that depressive, cyclothymic, irritable, and anxious temperament traits significantly elevate the risk of depression in hyperthyroid patients, with a particular emphasis on irritable temperament as a trigger for depressive symptoms. Conversely, hyperthymic temperament traits warrant assessment as a potential factor that may confer a protective effect against depression and enhance the mental resilience of patients. The results underscore the necessity of a multidisciplinary approach in hyperthyroidism management, extending beyond conventional treatment methods. Specialists, including endocrinologists, psychiatrists, clinical psychologists, primary care physicians, and nutritionists, should collaborate closely to ensure holistic patient management. In clinical practice, screening for temperament traits using validated tools such as the TEMPS-A may facilitate early identification of patients at risk, enabling timely psychotherapeutic intervention. Ensuring hormonal balance, alongside a careful evaluation of the individual's temperament traits and psychosocial structure, can enhance the effectiveness of the treatment process. The integration of personalized psychotherapeutic approaches with early diagnosis and intervention is essential for reducing depressive symptoms and enhancing the overall quality of life for patients.

Future long-term multicenter studies may clarify the mechanisms underlying these findings and contribute to the development of innovative treatment protocols that systematically incorporate psychiatric and psychosocial interventions, rather than concentrating exclusively on biochemical treatment in hyperthyroid patients. In contrast to the clear relationship found between depressive symptoms and temperament traits, our findings did not reveal a significant association between anxiety levels and specific temperament traits in hyperthyroid patients. This suggests that while temperament assessments can substantially inform depressive symptom management, their utility in predicting anxiety symptoms may be limited, necessitating further research. Therefore, an integrated approach to the endocrine and mental health of patients can enhance treatment outcomes and reduce the risk of relapse.
